# Warthin Tumor-Like Papillary Thyroid Carcinoma with a Minor Dedifferentiated Component: Report of a Case with Clinicopathologic Considerations

**DOI:** 10.1155/2010/495281

**Published:** 2010-06-07

**Authors:** Paolo Amico, Salvatore Lanzafame, Giovanni Li Destri, Paolo Greco, Rosario Caltabiano, Giada Maria Vecchio, Gaetano Magro

**Affiliations:** ^1^Section of Anatomic Pathology, Department G.F. Ingrassia, University of Catania, 95123 Catania, Italy; ^2^Department of Surgical Sciences, Organs Transplantation and Advanced Technologies, University of Catania, 95123 Catania, Italy

## Abstract

Warthin tumor-like papillary thyroid carcinoma is an uncommon variant of papillary thyroid carcinoma. We report a rare case of Warthin tumor-like variant of papillary thyroid carcinoma with a dedifferentiated component consisting of a solid tumor area composed of neoplastic cells with a spindle to tall cell morphology associated with marked nuclear pleomorphism, atypical mitoses, and foci of necrosis. Although our patient presented with a locally aggressive disease (T3 N1b Mo), she is disease-free without radioiodine therapy after a 23-month follow-up period. We emphasize that Warthin tumor-like papillary thyroid carcinoma, like other morphological variants of papillary carcinoma, may occasionally undergo dedifferentiation. As this component may be only focally detectable, we suggest an extensive sampling of all large-sized (>3 cm) papillary thyroid carcinoma. Recognition of any dedifferentiated component in a Warthin tumor-like papillary thyroid carcinoma should be reported, including its percentage, because it may reflect a more aggressive clinical course.

## 1. Introduction

Warthin tumor-like papillary thyroid carcinoma, first described in 1995 by Apel et al. [1], is a relatively uncommon variant of papillary thyroid carcinoma (PTC) with about 80 cases reported in the English literature to date [2–11]. The term “Warthin tumor-like PTC” was first coined on the basis of its close morphological resemblance to Warthin tumor, characteristically occurring in the salivary glands [1, 3, 4]. Clinically, it usually presents as a cystic or solid-cystic thyroid nodule [1–5]. Histologically it is composed of papillae lined by large, polygonal cells with abundant eosinophilic, finely granular cytoplasm, with a core exhibiting dense chronic inflammatory infiltrate, consisting predominantly of lymphocytes and plasmacells. Fine needle aspiration cytology (FNAC) and the histological examination of Warthin tumor-like PTC can pose diagnostic difficulties in distinguishing this neoplasm from a florid chronic thyroiditis, Hürthle cell nodules in chronic lymphocytic thyroiditis [11, 12], Hürthle cell tumors, tall cell and oncocytic variants of PTC, and, lastly, oncocytic variant of medullary carcinoma [12]. As far as differential diagnosis with lymphocytic thyroiditis are concerned, it is particularly intriguing that Warthin tumor-like PTC can be frequently documented in the context of Hashimoto thyroiditis [1–3, 11], representing a potential diagnostic pitfall. However, the main diagnostic criterion for diagnosis of Warthin tumor-like PTC is the detection of the typical nuclear features commonly seen in conventional type PTC, namely, optically clear nuclei, nuclear grooves, and intranuclear pseudoinclusions. Molecular biology studies have shown that Warthin tumor-like PTC and conventional PTC share the same BRAF and RET mutations, supporting that the former is a morphological variant of the latter [6]. However, whether Warthin tumor-like PTC should be considered a distinct clinico-pathologic entity with a favourable prognosis is still matter of debate [1, 3, 5, 7, 9], because some authors have reported that about 30% of cases exhibit a tendency to lymph nodal metastases and extrathyroidal extension [8, 9, 11]. The possibility of PTC to undergo dedifferentiation is a rare but well-known event which has a prognostic impact [4, 8, 13, 14]. Dedifferentiation usually consists of tumor solid areas characterized by plump spindle and squamoid cells with pleomorphic nuclei, in association with scattered areas of necrosis. High mitotic index and MIB-1 labelling index are usually associated features [8, 13]. A “tall cell” component can be also documented [8]. The presence of dedifferentiation in a PTC is usually associated with a worse prognosis, unless it is only focally detectable [8, 13, 14]. To the best of our knowledge, only one case of Warthin tumor-like PTC with a dedifferentiated component (10% of the entire tumor) has been reported in the literature [8]. As this tumour behaved aggressively, with diffuse infiltration of adjacent organs, distant metastases, and death of patient 18 months after thyroid surgery, the authors used the term “Warthin tumor-like variant of papillary thyroid carcinoma with dedifferentiation (anaplastic changes)” [8]. We herein report the clinico- pathological features of an unusual case of Warthin tumor-like PTC exhibiting a minor (about 5%) dedifferentiated component, consisting of marked nuclear pleomorphism, high mitotic index, atypical mitoses, tumor necrosis, and spindle to tall cell changes. Although our patient presented with a locally aggressive disease (T3 N1b M0), she is disease-free without radioiodine therapy after a 23-month follow-up period by means of clinical and imaging (TC) evaluation.

## 2. Patient Profile

A 79-year-old Caucasian woman presented at our observation for a swelling of the anterior neck. Clinical evaluation revealed a left thyroid mass and a swelling of the omolateral cervical region. Thyroid function blood tests revealed a high level of thyroglobulin protein (283 ng/ml), with no abnormalities for fT3, fT4, and TSH. An ultrasound was performed and showed a 52 × 47 × 61 mm solid-cystic mass, located in the left thyroid lobe and extending into mediastinum with deviation of the trachea. In addition, voluminous hypoechogenous nodular masses, suspicious for omolateral laterocervical lymph node metastases, were found.

## 3. Cytological Findings

**Figure 1:**
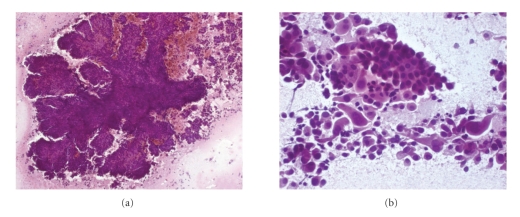
FNAC showing a large neoplastic papilla (a) and numerous mono- or bi-nucleated polygonal to spindle-shaped cells with abundant eosinophilic cytoplasm and large nuclei with finely dispersed chromatin and one or two prominent nucleoli; some cells show marked nuclear pleomorphism (b).

FNAC of the left thyroid mass was performed, showing several clusters and numerous papillae (Figure 1(a)) of polygonal to spindle-shaped cells with abundant eosinophilic cytoplasm and large nuclei with finely dispersed chromatin and one or two prominent nucleoli, suggestive of oncocytic cells (Figure 1(b)). Some of these cells showed marked nuclear pleomorphism and mitoses. Only rarely nuclear grooves could be identified. Some neoplastic cells were bi- or multinucleated. Numerous large-sized multinucleated giant cells and a small amount of lymphocytes were also present. FNAC of laterocervical masses showed red blood cells, granulocytes and clusters of oncocytic cells with mild atypia, in the absence of colloid and lymphoid cells. Cytological diagnosis was consistent with PTC, oncocytic variant, with lymph node metastases. Patient underwent total thyroidectomy and lymphadenectomy with laterocervical, recurrent nerve, and left jugular vein lymph nodes excision.

## 4. Gross Findings

**Figure 2:**
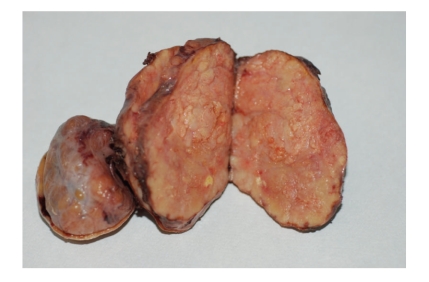
Gross examination. The left thyroid lobe is completely replaced by two nodules which, on cut section, are yellowish in colour.

Grossly, the left thyroid lobe was completely replaced by two nodules, measuring respectively 9 and 5 cm in greatest diameter, the former being solid and yellowish in colour (Figure 2), the latter solid-cystic with a central hemorrhagic area. Right thyroid lobe showed typical features of multinodular goiter.

## 5. Histological Findings

**Figure 3:**
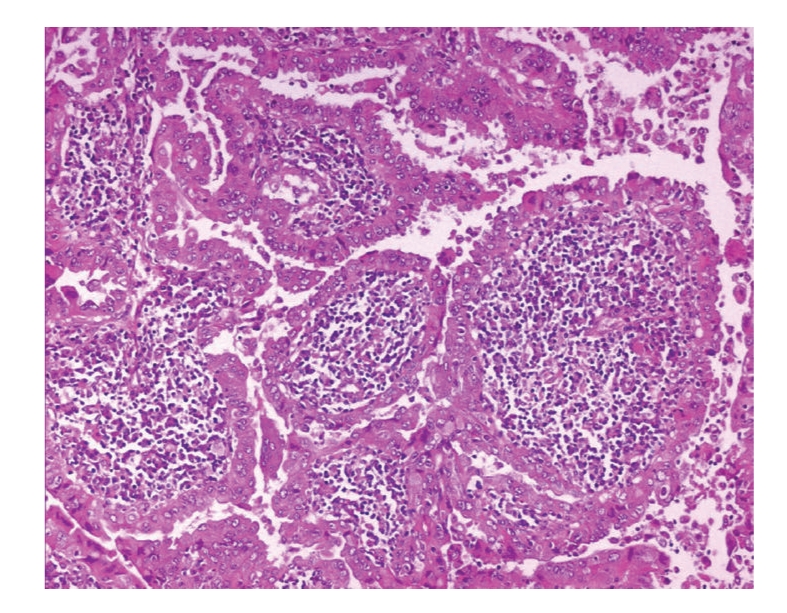
Histological examination of tumor showing the typical features of Warthin tumor-like PTC: papillae lined by oncocytic cells showing the typical nuclear features of PTC, and containing a dense inflammatory infiltrate, predominantly consisting of lymphocytes.

**Figure 4:**
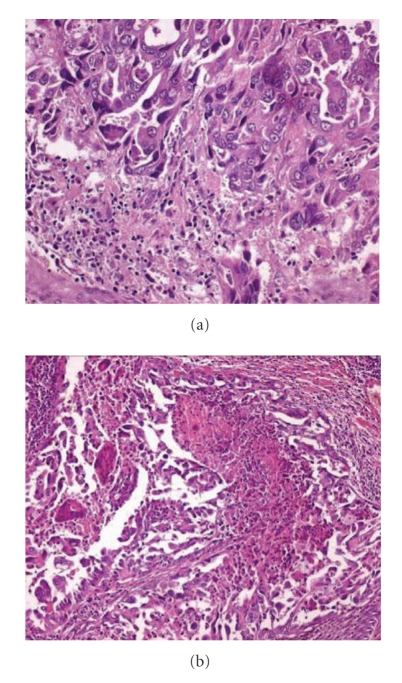
Dedifferentiation: tumor area composed of neoplastic cells with a spindle cell morphology and marked nuclear pleomorphism (a). Foci of tumor necrosis were scattered throughout the tumor (b).

**Figure 5:**
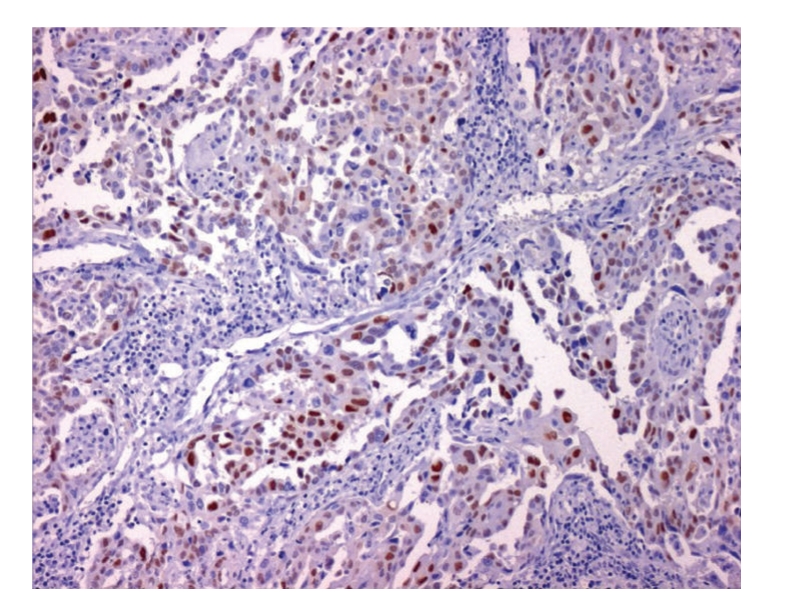
Most neoplastic cells showing a diffuse immunostaining with cyclin-D1.

Histological examination of left thyroid lobe revealed a tumor with a predominant papillary architecture. Tumor papillae were lined by oncocytic cells showing the typical nuclear features of PTC, namely, chromatin clearing, pseudonuclear inclusions, and grooves (Figure 3). The papillary stalks were entirely occupied by a dense inflammatory infiltrate, predominantly consisting of lymphocytes (Figure 3). Numerous multinucleated giant cells were also present. Notably, some tumor areas, with an overall diameter of 0.8 cm, had a solid growth pattern and were composed of neoplastic cells exhibiting marked nuclear pleomorphism, consisting of large-sized vescicular or hyperchromatic round to oval nuclei with one or more large nucleoli (Figure 4(a)). In addition some neoplastic cells showed both tall and spindle cell changes (Figure 4(a)). Mitotic activity ranged from 1 to 4 mitoses ×10 high-power field (HPF) and rare atypical mitoses were seen. Foci of tumor necrosis were also seen (Figure 4(b)). Tumor invaded thyroid capsule extending into perithyroidal adipose soft tissue. Immunohistochemically, neoplastic cells were diffusely and strongly stained with galectin-3, HBME-1, CK19, TTF-1, thyroglobulin, EMA, cytokeratins AE1/AE3, S-100 protein, and cyclin-D1 (Figure 5). The dedifferentiated component was positive to all above-mentioned antibodies and additionally to vimentin (focal expression). Focal expression of p53 was observed either in the classical tumour component or in the dedifferentiated component. The MIB-1 labelling index was about 2-3% in the classical oncocytic papillary thyroid carcinoma component and approximately 7% in the dedifferentiated component. Lymphocytes contained in the fibrovascular core of the papillae were represented by a mixture of T- and B-lymphocytes (CD3 + and CD20 +). Multinodular goiter was histologically confirmed in the right lobe. Ten regional lymph nodes, including omolateral laterocervical ones, were positive for metastasis. No tumour dedifferentiated component could be identified in lymph nodes metastases. Based on these pathological features, a diagnosis consistent with “Warthin tumor-like PTC with a minor (5%) dedifferentiated component” was rendered.

## 6. Discussion

Warthin tumor-like PTC is a relatively uncommon variant of PTC. The present study deals with a rare case of Warthin tumor-like PTC with a minor (5%) dedifferentiated component. Lam and colleagues in 2005, for the first time, reported a similar case with an aggressive biological behaviour and patient death [8]. Accordingly, these authors used the term “anaplastic changes” to describe the worrisome morphological features consisting, like in our case, of a solid tumor area characterized by spindle to ovoid to tall cells showing marked nuclear atypia, high MIB-1 labelling index (20%), and foci of necrosis. In our case, atypical mitoses were an additional finding of the dedifferentiated component. Interestingly, we showed that cyclin-D1 was overexpressed throughout the tumor, including dedifferentiated component. This finding would support previously published data suggesting that a high cyclin-D1 expression is usually associated with locally advanced disease in PTC [15–17]. Despite our case showed an overlapping morphology as compared to the previously described case by Lam et al. [8], our patient is well after a 23-month follow-up period from surgery. This apparently different clinical course may be explained by the fact that the patient reported by Lam et al., despite a FNA-based diagnosis of PTC, declined surgical treatment. Subsequently, she returned 3 years later with a significant increase in size of her thyroid mass and associated local symptoms due to diffuse tumor extension. It is likely that the minor (5%) dedifferentiated component of our case was not enough to impart tumor the ability to invade adjacent organs and/or distant metastases. On the contrary, we can speculate that the dedifferentiated component in the case of Lam et al. was not present at the time of cytological diagnosis, developing later, or alternatively, although it could be present at diagnosis, it increased over a period of three years, up to represent the 10% of the entire tumor component at surgery. We would like to emphasize that also Warthin tumor-like PTC, like other morphological variants of PTC, may occasionally undergo dedifferentiation and that this tumor component may be only focally detectable. Accordingly, we suggest that large-sized (>3 cm) Warthin tumor-like PTCs should be extensively sampled to exclude the presence of any dedifferentiated component. The present case, along with that previously reported [8], suggests that even a minor (5%–10%) dedifferentiated component in an otherwise typical Warthin tumor-like PTC has a prognostic impact. In fact both cases were presented with a locally advanced disease, that is, extrathyroidal extension plus lymph node metastasis and recurrent nerve involvement, respectively, in our case and in that by Lam et al. [8]. We suggest that the term “anaplastic changes” to indicate a dedifferentiated component in an otherwise specified Warthin tumor-like PTC should be abandoned to avoid confusion with true anaplastic carcinoma potentially arising from a PTC [13, 14]. The clinical evidence that our patient is disease-free after a 23-month follow-up from a radical surgical treatment, led us to prefer the term “Warthin tumor-like PTC with a minor dedifferentiated component” for such tumors. Lastly, we recommend that, like in typical PTC, the percentage of the dedifferentiated component should always be reported, because the survival rate of patient is likely dependent on its extension within tumor. Studies on large series could help to identify the exact size of the dedifferentiated component that reflects a worse clinical behaviour.
